# Antiosteoporosis and bone protective effect of dieckol against glucocorticoid-induced osteoporosis in rats

**DOI:** 10.3389/fendo.2022.932488

**Published:** 2022-08-19

**Authors:** Hao Wang, Leigang Yang, Junwei Chao

**Affiliations:** Department of Orthopedics, Xian Yang Central Hospital, Xianyang, China

**Keywords:** osteoporosis, dieckol, hormone, antioxidant, inflammation, bone turnover parameter

## Abstract

**Background:**

Glucocorticoids (GCs) induce osteoporosis, which results in fractures in the bond, causing significant morbidity. In the conducted study, we examined the antiosteoporosis effect of dieckol against GC-induced osteoporosis in rats.

**Methods:**

Sprague–Dawley (SD) rats were used for the current study and dexamethasone (2.5 mg/kg) induced osteoporosis in the rats that received the dieckol (test) and alendronate (standard) for 20 weeks. Bone turnover parameters, microCT, antioxidant, inflammatory cytokines, nutrient, and hormones parameters.

**Results:**

Dieckol noticeably suppressed the body weight and boosted the uterine and vagina weight. Dieckol considerably altered the level of trabecular number (Tb. N), the bone volume to total volume (BV/TV), trabecular separation (Tb.Sp), bone surface to bone volume (BS/BV), and t​r​a​b​e​c​u​l​a​r thickness (Tb.Th). Dieckol noticeably (P < 0.001) elevated the level of osteocalcin (OC) and alleviated the level of bone Gla protein (BGP), acid phosphatase (ACP), alkaline phosphatase (ALP), and β-CTx. Dieckol markedly boosted the level of malondialdehyde (MDA) and suppressed the level of glutathione (GSH), catalase (CAT), and superoxide dismutase (SOD) along with the suppression of inflammatory cytokines like TNF-α, IL-1β, and IL-6. Dieckol remarkably increased the level of calcium, potassium, magnesium, and 25 (OH) vitamin D. Dieckol substantially (P < 0.001) boosted the level of estradiol and alleviated the level of parathyroid hormone and tartrate-resistant acid phosphatase (TRAP). Dieckol also suppressed the level of receptor activator of nuclear factor κB ligand (RANKL) and boosted the level of osteoprotegerin (OPG).

**Conclusion:**

Taken together, our data suggest that dieckol demonstrated the anti-osteoporosis effect against GC-induced osteoporosis in rats.

## Introduction

Osteoporosis is a chronic bone disease characterized by decreased bone mass or bone mineral density (BMD), deterioration of bone architecture, and enhanced susceptibility to fracture. Osteoporosis disease is the most critical health issue after cardiovascular disease (1–3). The frequency of osteoporosis has increased worldwide. As per the report, approximately one in five men and one in three women suffered from osteoporosis, and also approximately 200 million people suffer from osteoporosis worldwide (4). The incidence of osteoporosis increased in elderly people (senile osteoporosis) and estrogenic deficiency induces osteoporosis in postmenopausal women (postmenopausal osteoporosis) (2, 5, 6). Furthermore, osteoporosis imposes a significant socioeconomic burden due to high rates of impairment, morbidity, and sometimes even death.

Osteoporosis is a chronic disease with nuanced pathophysiology (7). As per the previous reports, the pathology involved in osteoporosis is an alteration of bone remodeling (imbalance between osteoblast and osteoclast), which occurs due to the involvement of various factors such as inflammatory reaction and oxidative stress (7, 8). Various features are involved in osteoporosis like endocrine turmoil, nutritional value, and hereditary disarray (4). The currently available treatment for osteoporosis is selective estrogen receptor modulation, hormone replacement therapy (HRT), vitamin D (a vitamin supplement), calcium (Ca), magnesium (Mg), and phosphorus (P) (minerals) as well as anti-osteoporotic drugs such as zoledronic acid and bisphosphonates, immunosuppressants, 5-aminosalicylates, and corticosteroids (1, 9–11). However, all these therapies have limitations as they cause serious adverse effects such as kidney troubles; diarrhea; high blood pressure; breast, ovarian, or endometrial cancer; typical fracture; and stroke (12, 13). This research focuses on investigating alternative solutions that are more effective and have fewer side effects. Previous reports suggest that natural products with an antiosteoporosis effect, and their underlying cellular and molecular mechanisms arebeing extensively scrutinized (14, 15). It is well documented that vegetables, medicinal plants, and fruits can suppress the risk of osteoporosis in experimental rodents and humans (15–17).

Dieckol (phlorotannin), a phyto-constituent found in *Ecklonia cava* (family: Lessoniaceae), is a marine brown alga found in the oceans of Korea and Japan. Dieckol already proved its pharmacological effect against the various diseases due to its antioxidant potential (18, 19). Dieckol exhibited anti-ging, anti-inflammatory, anti-allergic, anti-diabetic, anti-hyperlipidemic, anti-neurodegenerative, and anti-tumor effects (19–23). Also, dieckol exhibited an anti-cancer effect against breast (24) and ovarian cancer (25). Dieckol exhibited an anti-cancer effect against various types of cancer due to its anti-cancer and anti-inflammatory effect. In this study, we scrutinized the antiosteoporosis effect of dieckol against the steroid-induced osteoporosis.

## Material and methods

### Animals

Sprague–Dawley (SD) rats (weight: 22–25 g; age: 5–6 months; sex: female) were obtained from the Department Animal House. The rats were housed in polyethylene cages under standard laboratory conditions (temperature: 215°C; relative humidity: 65%; and 12/12-h light/dark cycle). The rats were kept in the laboratory for 7 days prior to the experimental study.

### Induction of osteoporosis

For the induction of the osteoporosis, the subcutaneous injection of 2.5 mg/kg/day dexamethasone was administered continuously for 5 days (1).

### Test drugs

Dieckol (test drug) and alendronate (standard drug) were used to scrutinized the osteoporosis effect. Briefly, the suspension of dieckol was prepared using the 1% suspension of carboxymethyl cellulose (CMC).

### Experimental group

After the acclimatization of the laboratory condition, the rats were grouped and each group contained 10 rats.

Group A: Normal control;
Group B: GCs control;
Group C: Dieckol (50 mg/kg); and
Group D: Alendronate (20 mg/kg), respectively.

The drugs were selected based on the previously reported methods and, during the entire experimental protocol, the rats received one treatment of the test and the standard drug. The rats received the standard diet and water *ad libitum*. The body weights of all groups of rats were analyzed at regular time intervals. The rats received the administration of tetracycline and calcein and the animals were sacrificed 2 days later. All the rats were sacrificed, and sera, third lumber vertebrae, and femora were collected for further analysis.

### Organ weight

The organ weight is commonly used to analyze the degree of intuitive organ scratch and its morphological features. The vagina, uterus, and femur were removed and weighted before drying at 65°C to ensure an invariable weight, and the organ coefficients are thus estimated with slight variations to the previously disclosed method.

### Bone analysis

Bone parameters like BMD and BMC were measured at the end of the experimental study using the previous report with the help of Lunar Prodigy Dpxa-IQ-7040 (GE Heathcare, USA). The value was quantified through DEXA inbuilt software package.

### MicroCT

After sacrificing the rats, the right tibias were immediately removed. The bone tissue was kept and fixed well in sample cups and scanned. After being successfully scanned, the study selected the diaphyseal and inflated region of interest (ROI) of tibias. After the three-dimensional reconstruction threshold values were chosen, the ROI of the specimens was reconstructed. The bone volume (BV), total volume (TV), trabecula thickness (Tb.Th), bone volume/total volume (BV/TV), bone surface/bone volume (BS/BV), and trabecula separation (Tb.Sp) were scrutinized (1).

### Biochemical parameters

The blood sample of all groups was collected into the sterile test tubes. Afterward, the blood samples were allowed to clot, lysed, and finally centrifuged for 15 min at 5,000 rpm. After centrifuging the blood samples, the serum was separated and used to scrutinize the different biochemical markers like Mg, P, Ca, total cholesterol (TC), low-density lipoprotein (LDL), triglyceride (TG), high-density lipoprotein (HDL), and bone Gla protein (BGP). E2, ALP, ACP, MDA, CAT, SOD, GSH, and GPx were analyzed using the ELISA kit according to the information provided by the manufacturer (North Institute of Biological Technology, Beijing, China). The cytokine includes IL-6, TNF-α, and IL-1β was estimated using the ELISA kits in accordance to the manufacturer’s instructions.

### Statistical analysis

All of the relevant data were presented as means ± standard error means (SEM). One-way analysis of variance (ANOVA) was used for the estimation of statistical significance between each group. For the statistical analysis, GraphPad prism software version 5.0 for Windows (GraphPad Software Inc., La Jolla, CA, United States) was used, where P < 0.05 was considered significant.

## Results

### Body and organ weight

**Figure 1:**
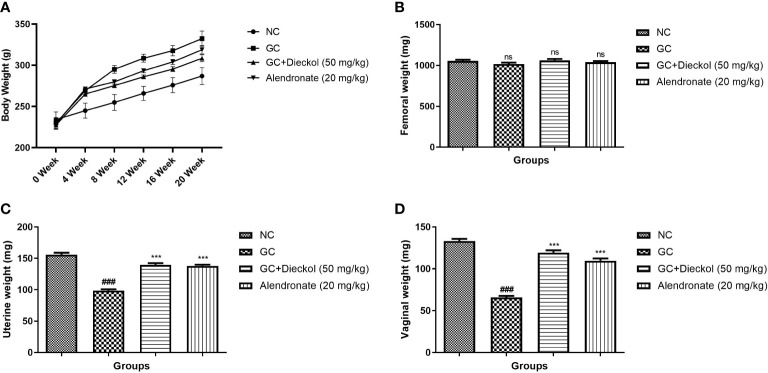
Dieckol showed an anti-glucocorticoid–induced osteoporosis effect **(A)** body weight, **(B)** femoral, **(C)** uterine, and **(D)** vaginal. Results are shown as means ± SEM (n = 12). ***P < 0.001 when contrasted with the disease group and the normal group.

In this investigation, we noticed the enhancement of the body weight after 4–5 weeks in the GC group (control) rats and dieckol treatment remarkably reduced the body weight (**Figure 1A**). The femoral weight of the GC group of rats slightly reduced, which was not significant. Dieckol-treated rats increased the femoral weight, but not significantly (**Figure 1B**). The GC group of rats exhibited the suppression of uterine and pancreas weight and dieckol treatment noticeably (P < 0.001) boosted the weight of uterine (**Figure 1C**) and pancreas (**Figure 1D**).

### MicroCT

**Figure 2:**
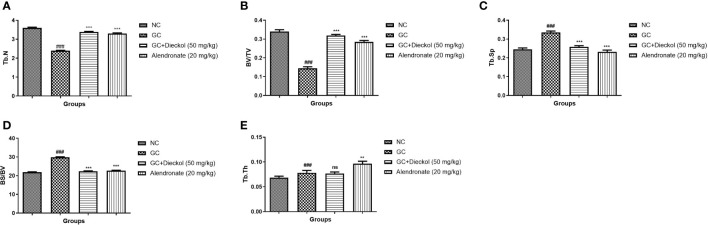
Dieckol showed an anti-glucocorticoid–induced osteoporosis effect on bone parameters in rats. **(A)** Tb.N, **(B)** BV/TV, **(C)** Tb.Sp, **(D)** BS/BV, and **(E)** Tb.Th. Results are shown as means ± SEM (n = 12). ***P < 0.001 when contrasted with the disease group and the normal group.

During osteoporosis, the level of microCT parameters was altered. In the study, the GC group exhibited the alteration in the level of microCT parameters and dieckol noticeably (P < 0.001) altered the level (**Figure 2**).

### Biochemical parameters

**Figure 3:**
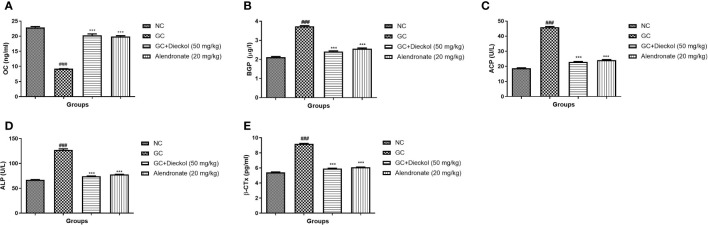
Dieckol was found to have an anti-glucocorticoid–induced osteoporosis effect on biochemical parameters in rats. **(A)** OC, **(B)** BGP, **(C)** ACP, **(D)** ALP, and **(E)** β-CTx. Results are shown as means ± SEM (n = 12). ***P < 0.001 when contrasted with the disease group and the normal group.

The rats in the GC-induced group had lower levels of OC (**Figure 3A**) and enhanced levels of BGP (**Figure 3B**), ACP (**Figure 3C**), ALP (**Figure 3D**), and β-CTx (**Figure 3E**) and dieckol significantly and alendronate markedly (P < 0.001) boosted the level of OC and lowered the level of BGP, ACP, ALP, and β-CTx.

### Estradiol, PTH, and TRAP

**Figure 4:**
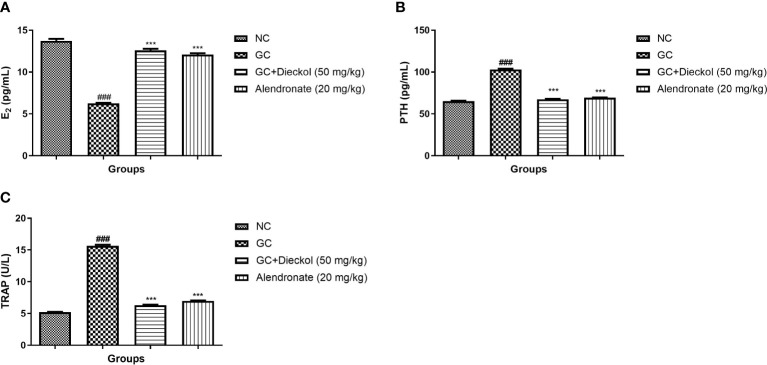
Dieckol inhibited glucocorticoid-induced osteoporosis in rats by increasing levels of estradiol, PTH, and TRAP. **(A)** E_2_, **(B)** PTH, and **(C)** TRAP. Results are shown as means ± SEM (n = 12). ***P < 0.001 when contrasted with the disease group and the normal group.

Estradiol levels were found to be decreased in the GC-induced group of rats (**Figure 4A**) and boosted level of PTH (**Figure 4B**) and TRAP (**Figure 4C**). Dieckol and alendronate noticeably (P < 0.001) boosted the level of estradiol and lowered the level of PTH and TRAP.

### Calcium, phosphorus, magnesium, and vitamin D

**Figure 5:**
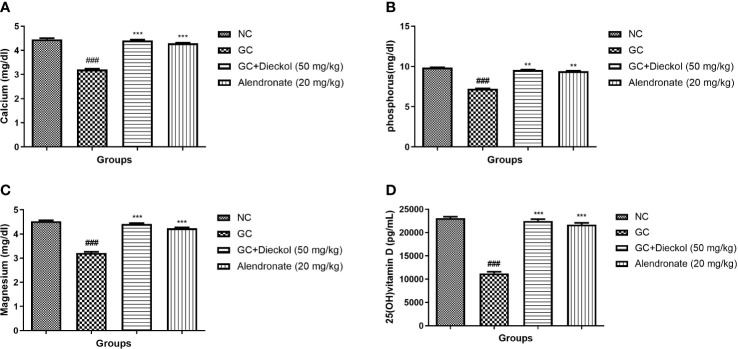
Dieckol was shown to protect nutrient parameters against glucocorticoid-induced osteoporosis in rats. **(A)** Calcium, **(B)** phosphorus, **(C)** magnesium, and **(D)** 25 (OH) vitamin **(D)** Results are shown as means ± SEM (n = 12). ***P < 0.001 when contrasted with the disease group and the normal group.

The GC group of rats showed the suppressed level of calcium (**Figure 5A**), phosphorus (**Figure 5B**), magnesium (**Figure 5C**), and vitamin D (**Figure 5D**). Dieckol and alendronate markedly (P < 0.001) boosted the level of calcium, phosphorus, magnesium, and vitamin D.

### Lipids

**Figure 6:**
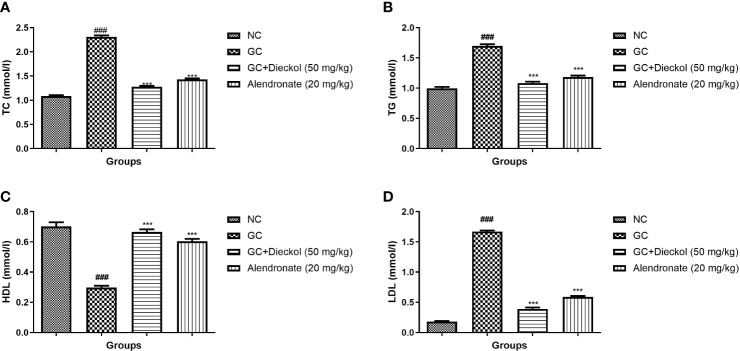
Dieckol was shown to protect lipid parameters against glucocorticoid-induced osteoporosis in rats. **(A)** TC, **(B)** TG, **(C)** HDL, and **(D)** LDL. Results are shown as means ± SEM (n = 12). ***P < 0.001 when contrasted with the disease group and the normal group.

The GC group of rats displayed the enhanced level of TC, TG, and LDL and decreased the level of HDL (**Figures 6A–D**, respectively). Dieckol and alendronate noticeably (P < 0.001) lowered the level of TC, TG, and LDL and elevated the level of HDL.

### Antioxidant

**Figure 7:**
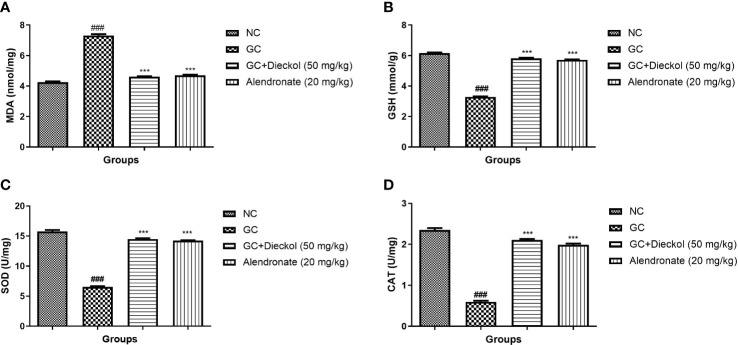
Dieckol inhibited glucocorticoid-induced osteoporosis in rats by increasing antioxidant parameters. **(A)** MDA, **(B)** GSH, **(C)** SOD, and **(D)** CAT. Results are shown as means ± SEM (n = 12). ***P < 0.001 when contrasted with the disease group and the normal group.

The GC group of rats exhibited elevated level of MDA (**Figure 7A**) and alleviated level of GSH (**Figure 7B**), SOD (**Figure 7C**), and CAT (**Figure 7D**). Dieckol and alendronate significantly (P < 0.001) decreased MDA levels, whereas GSH, SOD, and CAT levels had been increased. 

### Cytokines

**Figure 8:**
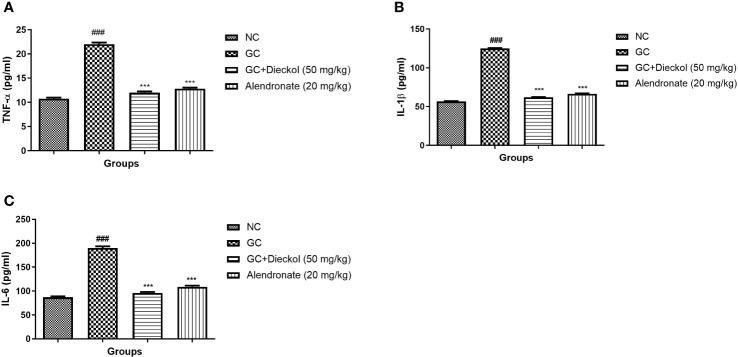
Dieckol was shown to have an anti-glucocorticoid–induced osteoporosis effect on cytokines in rats. **(A)** TNF-α, **(B)** IL-1β, and **(C)** IL-6. Results are shown as means ± SEM (n = 12). ***P < 0.001 when contrasted with the disease group and the normal group.

The inflammatory response is extremely crucial in the progression of osteoporosis. In the study, the GC group had a higher level of cytokines, whereas dieckol and alendronate significantly (P<0.001) alleviated the level of cytokines (**Figure 8**).

### RANKL and OPG

**Figure 9:**
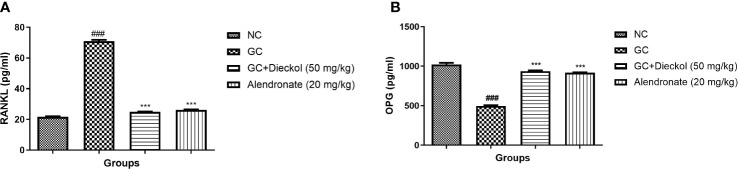
Dieckol was shown to have an anti-glucocorticoid-induced osteoporosis effect on RANKL and OPG parameters in rats. **(A)** RANKL and **(B)** OPG. Results are shown as means ± SEM (n = 12). ***P < 0.001 when contrasted with the disease group and the normal group.

The GC group demonstrated the elevated level of RANKL and reduced the level of OPG. Dieckol and alendronate markedly (P < 0.001) lowered the level of RANKL (**Figure 9A**) and enhanced the level of OPG (**Figure 9B**).

## Discussion

In this study, the level of OC altered during the osteoporosis due to the formation of osteoblast to make up for the bone loss caused by E2 deficiently. OC is the specific marker used to evaluate the bone turnover and formation. The patients undergo for the organ transplantation, and GCs drugs are commonly used for the treatment of gastrointestinal, autoimmune, and pulmonary disorders (26, 27). But long-term use of the GC exhibited the effect such as suppressing the bone density, which is the most common type of secondary osteoporosis after menopause (28). Osteoporosis clinical signs usually involve low bone density, deterioration of bone tissue, and disruption of bone micro-architecture, which together lead to an increased risk of fracture (29). Approximately 200 million people in the world have been diagnosed with osteoporosis and the chance of fracture increases with aging (4). Aside from fractures, it causes serious secondary health problems and mortality (26, 30). Dieckol is a marine polyphenol that exhibited various pharmacological effects against different diseases, including anti-inflammatory, anti-diabetic, and anti-cancer (19, 31–33). The osteoporosis effect of dieckol is still not explored; in this experimental research, we make effort to explore the antiosteoporosis effect.

The ongoing investigation discovered the shielding effect of dieckol against GC-induced osteoporosis in rats. Estrogen deficiency plays a key role in upholding the bone remodeling and mineralization mechanism *via* altering the equilibrium between the osteoclast and osteoblast (8). The body weight of the GC group of rats considerably boosted because of deficiency in relation with ovarian hormones especially estrogen. The current finding was supported by the previous report of Yang et al.,2021 (8) and dieckol remarkably suppressed the body weight of the GC group of rats

BMD and BMC are the significant parameters, commonly used for the estimation of bone quality and closely correlated to the osteoporosis degree (3). During osteoporosis, the level of BMD and BMC suppressed, a similar outcome was seen in the GS-induced group, and dieckol considerably suppressed the level of BMC and BMD.

OC is the significant biomarker of bone, which is commonly synthesized *via* osteoblasts and corresponds directly to the specific functions (7). Approximately a two-third part of OC was combined with the light apatite crystals and stored in the bone matrix and approximately a ^1^/_3_ part of OC enters in the bloodstream and shows the specific activity of osteoblasts. During the bone resorption, OC is released into the circulation (1). During osteoporosis, the level of OC boosted to dysregulation of bone formation and similar observation was found in our experimental study. These findings are in agreement with the results in the studies of An et al., 2021 and Mo et al., 2019 (1, 5). BGP initiates from the bone tissue, secreted, and synthesized *via* osteoblast in the bone (1, 3). BGP is the significant component of the bone non-collagenous proteins and approximately 3% of the bone contains the proteins. BGP plays a crucial role in the maintenance and regulation of bone calcium (1, 6, 11). ALP (phosphomonesterase) is commonly distributed in various organs such as the intestine, liver, lungs, and kidney (2, 6). Fifty percent of ALP is identified in the circulation and the rest are found in the hepatic tissue. The ALP is released from the osteoblast in the bone and deposited in the ossification sites like subperiosteum and epiphyseal line (1). During osteoporosis, the activity of bone parameters altered and, in the study conducted, dieckol remarkably reversed the effect. The result suggests that dieckol considerably suppresses the bone turnover and resorption to strengthen the bone formation and suppresses the bone loss.

It is well known that osteoporosis causes loss of bone and changes the structure due to alteration in the bone structure parameters. GC considerably suppressed the Tb.N and BV/TV parameters; Kamaruzzaman et al. (10) and Soelaiman et al. (34) found a similar result [10, 23]. In the study conducted, dexamethasone induced injury in the microstructure of proximal femoral bone *via* reducing BV/TV, Tb.Th, connD, and Tb.N and enhancing the structure model index and Tb.Sp. Due to the reduction of Tb.N, Tb.Sp, and BV/TV, after the dexamethasone treatment, the dangerous effect on the bone and its quality was exhibited and also altered the glucocorticoids signaling (10, 35). Dieckol treatment significantly restored the level of bone parameters suggesting the bone protective effect.

Reactive oxygen species (ROS) play a key role in the progression and expansion of osteoporosis disease (2, 36). ROS are mainly involved in the induction of estrogen deficiency and bone loss and also increase the osteoblast activity. During osteoporosis, the endogenous antioxidant system due to continuous generation of free radicals suppress. MDA (a lipid peroxidation marker) is strongly related with the oxidative stress. A previous report suggests that exposure of dexamethasone starts the production of superoxide radicals (O_2_), which as a result suppressed the activity of SOD and CAT (37, 38). The low activity of SOD, CAT and boost level of MDA, induces the oxidative stress, which further causing the injury in bone tissue. The results of the current investigation were in accordance with similar human osteoporosis trials (10). A study by Kamaruz zaman et al. (10) reported the reduction in the level of SOD and CAT and the enhancement in the level of MDA (10); the result was similar to our findings. Female rats suffering from osteoporosis had higher level of MDA and low level of GSH and GPx. Dieckol remarkably altered the level of antioxidant enzymes, which was modulated during the GC-induced osteoporosis.

Elbahnasawy et al. (39) suggested that osteocytes can alter the function and formation of osteoclasts and osteoblasts (39). Apoptotic osteocytes start the discharge of few inflammatory cytokines like TNF-α. TNF-α is involved in the stimulation of osteoclastogenesis (40). During the GCs treatment, initially, the osteocyte apoptosis starts and then the bone loss (8). In the study conducted, the dexamethasone group has the boosted level of inflammatory cytokines due to the induction of the apoptotic osteocytes. According to the result, dieckol remarkably suppressed the level of cytokines and suggested the anti-inflammatory effect.

RANKL and OPG are the two crucial cytokines expressed *via* osteoblasts and play a crucial role in bone metabolism (5). RANKL plays a critical role in the bone resorption *via* combining with its receptor during the bone metabolism (5, 41). In the study, we observed the reduced level of OPG and boosted level of RANKL, which was similar to the previous reports (5). Dieckol significantly altered the level of OPG and RANKL and suggested the bone protective effect.

## Conclusion

In summary, dieckol remarkably suppressed the body weight and elevated the uterine and vagina weight, considerably altered the bone microstructure, hormones, and nutrients, and improved the level of endogenous antioxidant and suppressed the inflammatory reaction. Therefore, dieckol could be used as a prophylactic agent to protect the glucocorticoid-induced osteoporosis.

## Data availability statement

The original contributions presented in the study are included in the article/supplementary material. Further inquiries can be directed to the corresponding author.

## Ethics statement

All the animal experiments were performed in line with the Xian Yang Central Hospital guidelines using approved procedures of the Institutional Animal Care and Use committee at Xian Yang Central Hospital.

## Author contributions

HW performed the experimental study. LY and JC interpretate the experimental data. JC design the study. All authors equally contributed in drafting and proof reading. 

## Conflict of interest

The authors declare that the research was conducted in the absence of any commercial or financial relationships that could be construed as a potential conflict of interest.

## Publisher’s note

All claims expressed in this article are solely those of the authors and do not necessarily represent those of their affiliated organizations, or those of the publisher, the editors and the reviewers. Any product that may be evaluated in this article, or claim that may be made by its manufacturer, is not guaranteed or endorsed by the publisher.
